# Positron Emission Tomography (PET) and Neuroimaging in the Personalized Approach to Neurodegenerative Causes of Dementia

**DOI:** 10.3390/ijms21207481

**Published:** 2020-10-11

**Authors:** Maria Ricci, Andrea Cimini, Agostino Chiaravalloti, Luca Filippi, Orazio Schillaci

**Affiliations:** 1 Department of Biomedicine and Prevention, University of Rome Tor Vergata, 00133 Rome, Italy; andreacimini86@yahoo.it (A.C.); agostino.chiaravalloti@uniroma2.it (A.C.); orazio.schillaci@uniroma2.it (O.S.); 2 Nuclear Medicine Section, IRCCS Neuromed, 86077 Pozzilli, Italy; 3 Nuclear Medicine Section, “Santa Maria Goretti” Hospital, 04100 Latina, Italy; lucfil@hotmail.com

**Keywords:** Alzheimer’s disease, dementia, frontotemporal dementia, alpha-synucleinopathies, FDG

## Abstract

Generally, dementia should be considered an acquired syndrome, with multiple possible causes, rather than a specific disease in itself. The leading causes of dementia are neurodegenerative and non-neurodegenerative alterations. Nevertheless, the neurodegenerative group of diseases that lead to cognitive impairment and dementia includes multiple possibilities or mixed pathologies with personalized treatment management for each cause, even if Alzheimer’s disease is the most common pathology. Therefore, an accurate differential diagnosis is mandatory in order to select the most appropriate therapy approach. The role of personalized assessment in the treatment of dementia is rapidly growing. Neuroimaging is an essential tool for differential diagnosis of multiple causes of dementia and allows a personalized diagnostic and therapeutic protocol based on risk factors that may improve treatment management, especially in early diagnosis during the prodromal stage. The utility of structural and functional imaging could be increased by standardization of acquisition and analysis methods and by the development of algorithms for automated assessment. The aim of this review is to focus on the most commonly used tracers for differential diagnosis in the dementia field. Particularly, we aim to explore ^18^F Fluorodeoxyglucose (FDG) and amyloid positron emission tomography (PET) imaging in Alzheimer’s disease and in other neurodegenerative causes of dementia.

## 1. Introduction

Dementia is defined as a disorder that leads to a significant decline in cognitive functions and that causes interference in daily living, including occupational, domestic, or social functioning. Generally, dementia should be considered an acquired syndrome that may be due to multiple possible causes. Global estimates of dementia prevalence are 5–7% of individuals above the age of 60 in most world regions, with a slightly higher prevalence in developed countries due to longer life spans and a distinctively lower prevalence in the four sub-Saharan African regions (2–4%) [1]. Dementia may be due to non-neurodegenerative causes, including vitamin deficiencies (e.g., B12, thiamine) or systemic diseases such as hypothyroidism, normal pressure hydrocephalus, and infections. Moreover, common non-neurodegenerative causes include chronic alcohol abuse, chemotherapy-related cognitive dysfunction, and psychiatric illness or brain alterations such as intracranial masses (e.g., subdural hematomas, brain tumors) or traumatic brain injury [2]. Therefore, an accurate global examination is mandatory for differential diagnosis in order to exclude non-neurodegenerative causes. This review aims to focus on differential diagnosis of the neurodegenerative or mixed causes of dementia. Neuroimaging is an essential tool for differential diagnosis of multiple causes of dementia and allows a personalized diagnostic. The therapeutic protocol is based on risk factors that may improve treatment management, especially in early diagnosis during the prodromal stage, by using novel algorithms for automated assessment and statistical analysis.

## 2. Alzheimer’s Disease (AD)

Alzheimer’s disease is the most common neurodegenerative dementia from middle age to the elderly, with a prevalence of 5–6% of all individuals over the age of 65, reaching 30% in those over the age of 85 [3]. The pathophysiology of AD is increasingly becoming clearer through numerous research papers concerning postmortem evaluation, genetic risk, biochemical interaction, and primarily structural and functional neuroimaging. Functional neuroimaging is considered an essential tool in the diagnosis of AD and is also included in diagnostic criteria for the most prevalent non-Alzheimer’s dementias, reflecting its value both in the differential diagnosis of AD and in the personalized therapeutic management of patients [4]. The most validated biomarkers for AD fall under two categories: those that reflect the specific pathology of AD and those that reflect neuronal damage or dysfunction. The pathological biomarkers of AD are amyloid-beta and tau imaging and a cerebrospinal fluid assay of the amyloid peptide and tau protein. Atrophy, according to Braak staging [5], can be a valuable marker of neurodegeneration, particularly atrophy of medial temporal structures, as measured with structural magnetic resonance imaging (sMRI) [6].

Nevertheless, the purpose of most research papers is to focus on an even earlier diagnosis before an irreversible neuronal loss occurs in order to maximize the therapeutic impact on clinical aspects [7]. It remains challenging to establish reliable markers for diagnosing and monitoring disease progression in the early stages and on an individual basis. Several AD biomarkers are used as outcome measures in trials of potentially disease-modifying therapies [6], which include mostly functional neuroimaging biomarkers.

### 2.1. FDG Imaging in AD

In vivo, functional neuroimaging has long been used to evaluate brain functional abnormalities such as biomarkers and predictors of AD in the elderly. The most widely utilized positron emission tomography (PET) tracer for AD in clinical practice is FDG, which measures the underlying neuronal activity [8], although the use of Aβ and tau tracers for positron emission tomography is rapidly growing. The role of functional imaging is growing because metabolic alterations are hypothesized to precede the appearance of cognitive symptoms in AD. Therefore, FDG-PET imaging is considered a useful biomarker for investigating the metabolism changes triggered by AD neurological changes across the entire AD clinical continuum, ranging from the presymptomatic phase to the stage of dementia [9]. FDG-PET research papers describe a characteristic bilateral temporoparietal hypometabolism in AD and show the accuracy of brain FDG-PET in discriminating between patients with mild cognitive impairment (MCI) due to Alzheimer’s disease and healthy controls, especially evaluating predefined anatomical-functional regions and inter-hemispheric asymmetries [10]. Moreover, FDG-PET imaging may allow the identification of AD subgroups with clinical implications: about 5% of all Alzheimer’s disease occurs before age 65, which is conventionally termed “early-onset” [11] and shows a different metabolic pattern in FDG-PET imaging with respect to “late-onset” and different clinical findings [12]. 

EANM-EAN (European Association of Nuclear Medicine and European Academy of Neurology) recommendations for the use of brain ^18^F-fluorodeoxyglucose positron emission tomography (FDG-PET) in neurodegenerative cognitive impairment and dementia proposed the use of FDG-PET in the case of MCI conditions, in order to define the neurodegenerative disorder underlying the cognitive condition [13]. This included the conditions of AD, frontotemporal dementia (FTD), and dementia with Lewy bodies (DLB). Notably, the panel voted for FDG-PET to support the diagnosis of AD in MCI because FDG-PET allows a better short-term prognosis of AD dementia conversion as compared to biomarkers of amyloidosis, and may also identify non-Alzheimer’s types of neurodegeneration early in the course of the disease. FDG-PET in subjective cognitive decline, in asymptomatic subjects at risk for AD and in asymptomatic subjects with familial forms of AD, even if its role in genetic polymorphism is supported in the literature [14], according to the panel, should be regarded only within well-defined, ethics committee-approved research studies, but definitively not in clinical practice. The use of FDG-PET is supported to facilitate the differential diagnosis between different forms of dementia and AD (particularly in patients with atypical presentation of atypical course): between AD and DLB, between AD and FTD (frontotemporal dementia), or between AD and vascular dementia [13,15]. 

FDG-PET may have a potential role in the assessment of the response to treatment in patients with AD, detecting the effects of therapies on cerebral glucose metabolism. Shimada et al. [16] evaluated eleven patients with AD dementia, finding significant differences in the glucose metabolism of the occipital and frontal lobes between responders and non-responders to therapy with an acetylcholinesterase inhibitor (donepezil). Mega et al. [17] demonstrated a significant improvement in the prefrontal cortex glucose metabolism of AD patients who were responders to therapy with galantamine, a cholinesterase inhibitor and a nicotine receptor modulator. Furthermore, an exciting study by Wang et al. investigated the effects on glucose metabolism of memantine in patients with AD: in comparison to the placebo group, the authors demonstrated higher values of regional cerebral metabolic rate for glucose in patients receiving memantine, especially in regions affected by the disease [18]. The effects of memantine on cerebral glucose metabolism in patients with AD mainly concern an increase in metabolic activity in the parietal and temporal lobes, as suggested in a study by Sulzter et al. [19].

### 2.2. Amyloid Imaging in AD

In vivo, amyloid-beta imaging with PET is a validated diagnostic tool for detecting fibrillar amyloid-beta, particularly neuritic plaques and amyloid angiopathy. Pittsburgh compound B (PIB) is a radioactive analog of thioflavin T used in histopathology, which crosses the blood–brain barrier and binds to amyloid plaques with high affinity to specifically insoluble fibrillary forms of amyloid-beta [20], detecting particularly neuritic plaques and amyloid angiopathy [21,22]. Moreover, amyloid-beta PET ^18^F-labeled tracers have been developed (florbetaben, florbetapir, flutemetamol) [23] with high sensitivity and specificity in vivo [24] and in vitro [25,26]. Amyloid imaging, in particular, is already being used as part of the eligibility criteria in large, multicenter clinical therapeutic trials [27]. In vivo amyloid imaging reveals differences in amyloid burden in various brain regions in different subject groups, with higher amyloid burden in AD and MCI subjects than in healthy individuals [7], confirmed by the correlation between amyloid PET findings and the gold-standard postmortem histopathology [28]. Much of the literature on amyloid PET focuses on patients with mild cognitive impairment (MCI), as the common belief is that this is the stage in disease progression where meaningful intervention can be made. Several studies have found correlations between patients with MCI who are positive to in vivo amyloid evaluation on PET (both PIB and ^18^F-labeled tracers) and their rates of conversion to AD on follow-up [29,30]. In addition to its clinical utility, amyloid PET imaging has revolutionized therapy development for AD. Amyloid PET scans have been used with success as part of the inclusion criteria for enrolling patients in promising trials. Although these trials have so far produced disappointing results in terms of cognitive benefit, some have demonstrated a modest reduction in cerebral amyloidosis and encouraging clinical results [31,32]. Amyloid PET imaging may have a role in the monitoring of therapy with monoclonal antibodies such as crenezumab or gantenerumab: these monoclonal antibodies bind with high affinity to amyloid-beta peptides, promoting their removal. PET with ^18^F-florbetapir was used in order to assess the reduction in amyloid load in two randomized studies [33,34]. Furthermore, amyloid PET was used by Egan et al. [35] in order to assess the amyloid burden in patients with memory impairment receiving oral verubecestat, a β-site amyloid precursor protein-cleaving enzyme 1 inhibitor.

**Figure 1:**
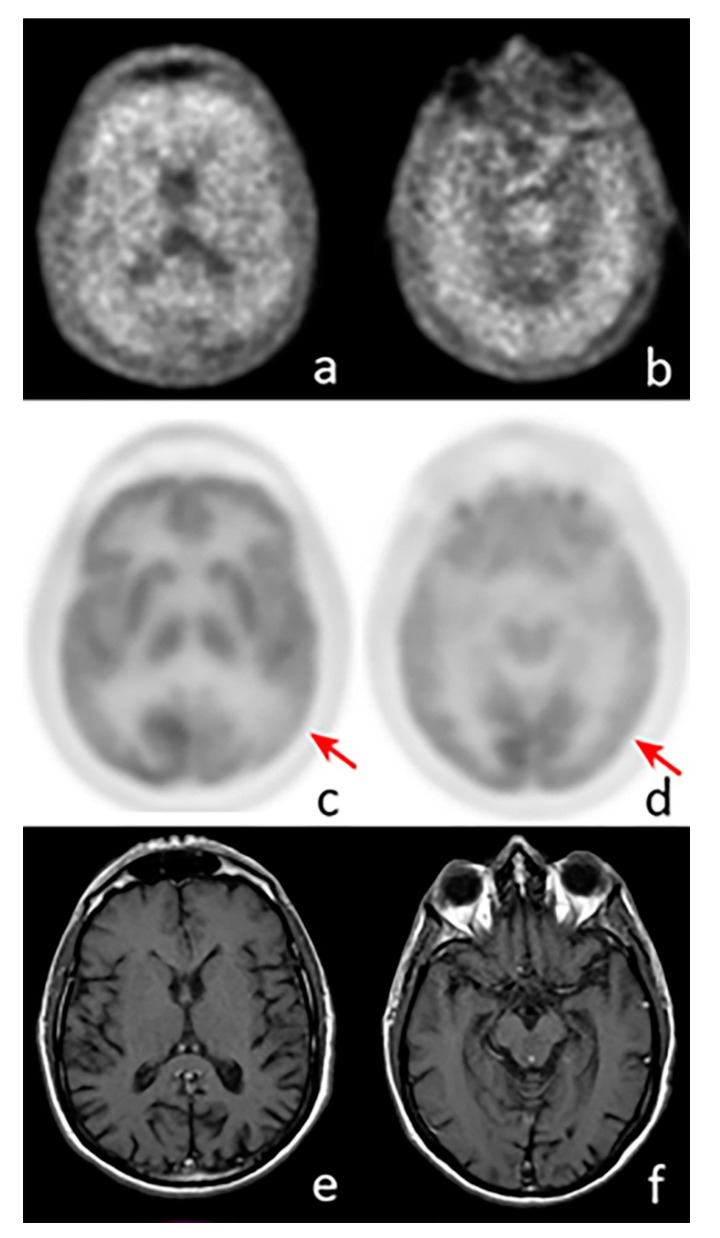
Axial images of a positron emission tomography (PET) scan performed with ^18^F-Florbetaben (**a**,**b**), ^18^F-Fluorodeoxyglucose (**c**,**d**), and MRI (**e**,**f**) in a patient with suspected AD. The images show pathological amyloid burden in the cortex, while a significant reduction in cortical glucose consumption is visible in the left parietal (c, arrow) and temporal lobes (d, arrow). MRI does not show any abnormal findings.

**Figure 2:**
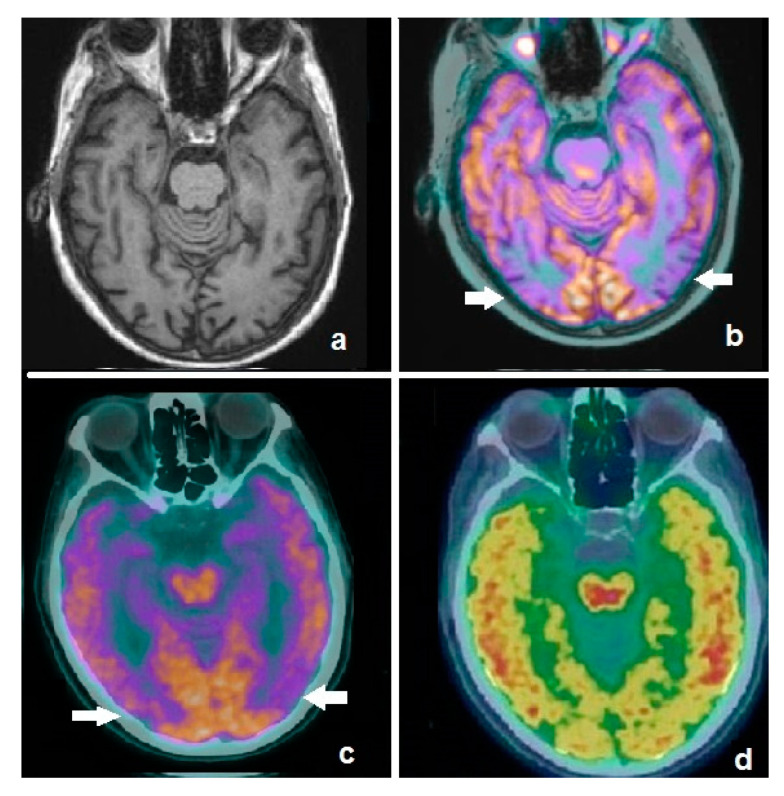
Axial image of an MRI scan in a patient affected by mild cognitive impairment (**a**), showing no significant abnormalities. Fused corresponding ^18^F-FDG PET/MRI demonstrates hypometabolism in temporo-occipital lobes (**b**, white arrows). Patient was submitted to dual-phase amyloid PET with ^18^F-flutemetamol: the early phase demonstrates an AD-typical pattern (**c**, white arrows), substantially overlapping with the ^18^F-FDG findings, while the late phase shows pathological amyloid burden in the cortex (**d**). Dual-phase amyloid PET resulted to be useful for the simultaneous detection of neuronal dysfunction and amyloid burden.

Of note, amyloid PET and FDG-PET provide complementary information in AD (Figure 1), since the former reflects the load of amyloid deposits and the latter the entity of metabolic disruption; therefore, a single imaging modality capable of simultaneously assessing both of them would be of great usefulness. It has been reported, in fact, that the co-occurrence of neuronal dysfunction and amyloid deposits in cognitively normal individuals represents a significant risk factor of developing a decline in cognitive functions over time [36]. The aforementioned considerations led to the development of a peculiar technique of acquisition, termed “dual-phase amyloid PET”, consisting in an early phase (5–6 min) acquired immediately after the administration of the radiocompound (i.e., PIB or ^18^F-labeled tracers) with the aim of assessing brain perfusion, followed by a late phase to evaluate amyloid deposits [37,38]. Several research studies indicate that the early phase of amyloid PET provides information substantially overlapping with that obtained from perfusion brain single-photon emission CT (SPECT) or FDG-PET, reflecting neuronal injury. In a recently published paper by Florek et al., the role of dual-phase amyloid PET was investigated in 112 patients (41 with MCI, 50 with probable/possible AD, 21 with other dementias) submitted to ^18^F-Florbetaben (FBB) PET. Among the enrolled subjects, visual analysis of the early phase revealed an AD-typical pattern in 39% of cases, while in patients with dementia due to AD and MCI due to AD, an AD-typical perfusion pattern was found in 42% and 27% of patients, respectively [39]. These preliminary results highly encourage further clinical investigations on dual-phase amyloid PET, which might result to be of particular usefulness to identify asymptomatic/pauci-symptomatic subjects who are more likely to benefit from upcoming prevention clinical trials (Figure 2).

### 2.3. Tau Imaging in AD

Tau PET imaging allows the in vivo evaluation of tau protein distribution, the primary component of neurofibrillary tangles. PET imaging of Neurofibrillary tangles (NFTs) holds promise not only as a diagnostic tool but also because it may enable the development of disease-modifying therapeutics for dementia [40]. Concerning in vivo tau imaging’s role in AD, the non-invasive assessment of the spatial and temporal pattern of tau deposition over time may provide an insight into the role tau plays in AD and may lead to establishing the relation between cognition, genotype, neurodegeneration, and other biomarkers in AD. The topographic distribution of tau tracers (^18^F-AV-1451, ^18^F-THK535149, and ^11^C-PBB3) in amnesic MCI or AD dementia, compared with normal aging, is consistent with Braak staging, with more prominent tracer binding in inferior and lateral temporoparietal cortices, parieto-occipital cortices, posterior cingulate cortices, and the precuneus and less prominent in frontal regions and primary sensorimotor cortices [41,42,43]. Moreover, it is essential to underline the relationship between tau spreading and disease progression [44]: therefore, once validated in clinical practice, selective tau imaging might be useful as a diagnostic, prognostic, and progression biomarker and as a surrogate marker for the monitoring of efficacy and patient recruitment for anti-tau therapeutic trials [45].

## 3. Frontotemporal Dementia (FTD)

Frontotemporal dementia (FTD) is an “umbrella term” that includes a cluster of neurocognitive syndromes characterized by an impairment of executive functioning, including changes in behavior and alterations in language capability. After the most prevalent form AD, FTD is the next most common form of dementia in the population younger than 65 years, and it is expected to increase in prevalence with the increase of the population mean age [46]. Functional imaging studies, such as single-photon emission CT (SPECT) and ^18^F-fluorodeoxyglucose positron emission tomography (FDG-PET), typically demonstrate substantial abnormalities, while amyloid imaging is not commonly used. Moreover, tau imaging seems to be a promising tool, while PET with dopaminergic (e.g., ^18^F-fluoro-L-dopa (6FD) and ^11^C-raclopride) tracers reveals uptake abnormalities different from those of Parkinson’s disease (PD) [47].

### 3.1. FDG-PET in FTD

Functional neuroimaging with FDG-PET is used to confirm the clinical hypothesis regarding the localization of neurological changes, which may lead to the differential diagnosis of FTD with AD, DLB, or pathologies most often underlying the condition [41]. The diagnosis of FTD may be a challenge, especially in the prodromal stage, where behavioral changes may mimic psychiatric disorders and cognitive impairment is absent or may present as mild behavioral impairment. Because frontotemporal hypometabolism can be detected in the prodromal stage when the patient presents with the first cognitive symptoms, or behavioral symptoms, FDG-PET is required by current diagnostic criteria for probable FTD at the dementia stage [13].

The use of FDG-PET is supported to facilitate differential diagnosis among different primary forms of dementia, especially between FTD and AD; occasionally, differentiating FTD from AD on clinical-neuropsychological grounds may be challenging. In most cases, the hypometabolic patterns of FTD and AD are separated. In FTD, the hypometabolism mainly involves the prefrontal, insular and anterior cingulate cortex, basal ganglia, and sometimes the crossed cerebellar diaschisis; while in AD, the characteristic hypometabolic pattern mainly involves the posterior cingulate cortex and precuneus. Nevertheless, frontoparietal hypometabolism may sometimes be found in both diseases, which may also coexist [13]. PET-FDG may be indicated in the differential diagnosis between DLB and FTD. FTD shows frontal and anterior-temporal hypometabolism, and DLB displays additional posterior involvement (visual and parieto-temporal) and relative posterior cingulate preservation, with some overlapping features, particularly relevant in the presence of parkinsonism [13,48,49].

**Figure 3:**
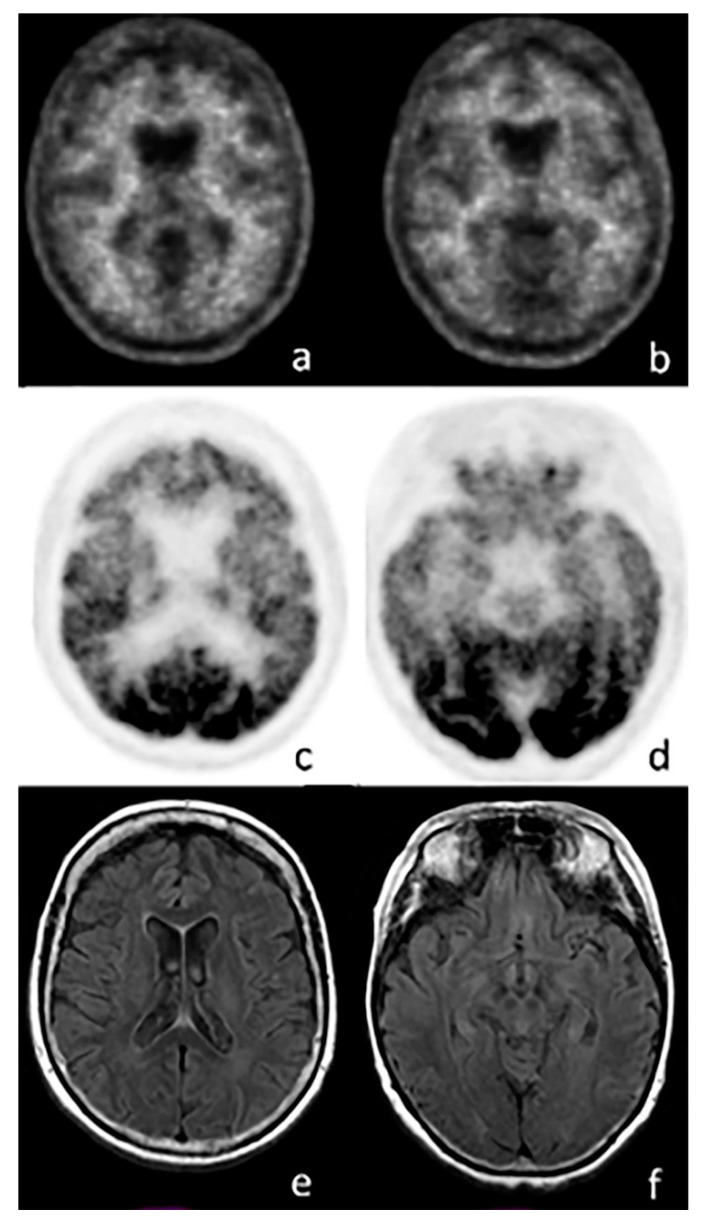
Axial images of a PET scan performed with ^18^F-FBB (**a**,**b**), ^18^F-FDG (**c**,**d**), and MRI (**e**,**f**) in a patient with suspected frontotemporal dementia (FTD). The images show no amyloid burden in the cortex, while a significant reduction in cortical glucose consumption is visible in the frontal (**c**) and temporal lobes (**d**). MRI does not show any abnormal findings.

FDG-PET is used in clinical practice (Figure 3) for the differential diagnosis with corticobasal syndrome [13]. Hypometabolism is generally localized in motor and premotor cortices, but can also involve the prefrontal or posterior parietal and lateral temporal cortex and the cingulate gyrus. Moreover, basal ganglia and thalamus present hypometabolism in the same hemisphere, harboring cortical hypometabolism. This heterogeneity is consistent with the variety of diseases causing CBS, which mainly include CBD (corticobasal disease), progressive supranuclear palsy (PSP), AD, FTLD, or mixed pathologies. In cases of anterior cingulate and associated frontal cortex hypometabolism, PSP or FTD is suspected, whereas basal ganglia hypometabolism supports the diagnosis of CBD and PSP [13].

Similarly to AD, FDG-PET may be useful in the evaluation of the response to therapy in patients with FTD, especially those receiving memantine in which an increase in metabolic activity in the insulae and orbitofrontal cortex after 7–8 weeks of treatment was demonstrated [50].

### 3.2. Amyloid Imaging in FTD

Amyloid PET imaging usually displays no tracer retention in patients with the clinical syndrome of FTD [51,52] (Figure 3); therefore, it can be used in the differential diagnosis in order to exclude AD when the suspected underlying pathology is FTD, especially in cases clinically manifesting as behavioral variant FTD (bvFTD) or PPA (Primary Progressive Aphasia). In fact, in the case of a patient that presents clinical features of FTD and a positive amyloid imaging, the diagnosis may be AD or mixed pathologies (coexisting FTD and AD that both contribute to dementia), or the positive scan may be interpreted as an incidental finding (therefore relatively low neurofibrillary alteration that is not contributory to symptoms) [41]. The use of amyloid PET imaging is considered most appropriate when patients present with cognitive impairment that could be attributed to AD. Nevertheless, where the clinician is uncertain, the confirmation of presence or absence of amyloid may influence the diagnosis [41,53,54,55].

### 3.3. Tau Imaging in FTD

In the past few years, several studies have demonstrated the potential uses of an imaging biomarker that evaluates in vivo tau retention, which represents a promising, useful biomarker not only for AD but also for other forms of dementia that presents tauopathies, including FTD [41]. In vivo tau PET imaging presents opportunities to improve both clinical practice and research for the spectrum of FTD, including primary tauopathies as well as TDP-43 proteinopathies. Preliminary studies have shown that ^18^F-AV-1451 increased uptake in the frontal and temporal cortices of patients with FTD, a pattern expected for the distribution of tauopathy in FTD. In addition, other tracers are being investigated in order to evaluate the tauopathy in the FTD spectrum as well, including ^18^F-THK5351 [41,47,56].

## 4. The Alpha-Synucleinopathies (SNCApathies)

**Figure 4:**
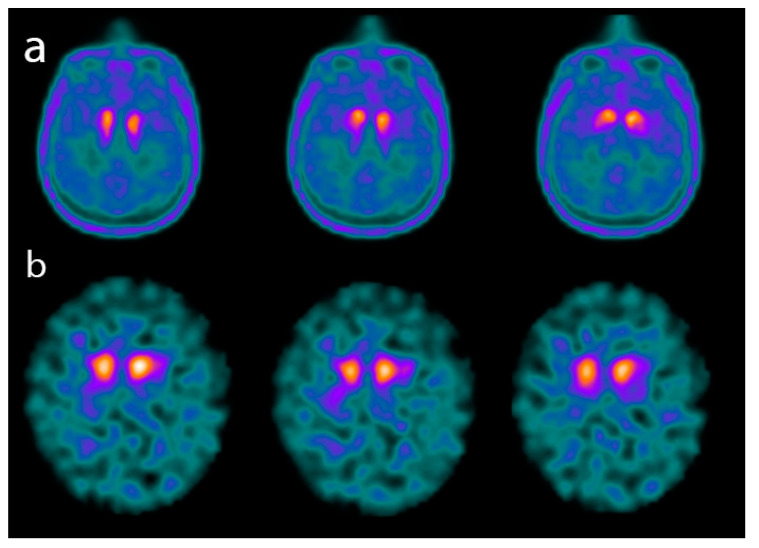
Axial images of a PET scan performed with ^18^F-Fluoro-L-dopa (FDOPA) (**a**) and of a single-photon emission CT (SPECT) scan performed with ^123^I-FPCIT (DATSCAN) (**b**) in two patients affected by advanced dementia with Lewy bodies (DLB). The images show a significant reduction in both ^18^F-FDOPA and ^123^I-FPCIT uptake in the basal ganglia.

The alpha-synucleinopathies (SNCApathies) are a group of several neurodegenerative diseases that are characterized by the pathological accumulation of alpha-synuclein aggregates in neurons and cells of the Central nervous system (CNS), including dementia with Lewy bodies (DLB), Parkinson’s disease (PD), or multiple system atrophy (MSA). Clinical features of DLB include fluctuating (typically delirium-like and occurring as spontaneous alterations in cognition, attention, and arousal); recurrent complex visual hallucinations; sleep behavior disorder during rapid eye movement sleep; one or multiple spontaneous features of parkinsonism (bradykinesia, tremor at rest, rigidity) [2,57]. Nevertheless, parkinsonism is common among many dementias and may be due to neurodegenerative pathology, as in the alpha-synucleinopathies, corticobasal syndrome, and progressive supranuclear palsy, or due to a secondary effect of other brain alterations injuries [57]. In PD dementia (PDD), similar symptoms have been described but follow at least after 1 year of well-established PD onset [2], whereas in DLB the cognitive decline may be antedated or occur simultaneously. MSA, the most rapidly progressive of the alpha-synucleinopathies, is a rarer disease that manifests with any combination of parkinsonism, cerebellar signs, pyramidal signs, and dysautonomia symptoms common also in further dementia-related disease. In the clinical setting, the alpha-synucleinopathies are often misdiagnosed. Aggregation of α-synuclein (SNCA) in Lewy bodies and neurites often coexists with amyloid-β plaques and tau neurofibrillary tangles; therefore, an integrated neuroimaging approach is a fundamental tool in the diagnosis of DLB [58] (Figure 4).

### 4.1. Dopamine Transporter (DAT) Imaging in SNCApathies

Severe nigrostriatal dopaminergic degeneration occurs in alpha-synucleinopathies, but not in AD or further dementia subtypes. Pre- and postsynaptic dopaminergic neuronal function, as assessed with nuclear imaging techniques, has been widely documented in parkinsonian conditions. Multiple tracers are available, including PET tracers such as ^18^F-dopa (dopamine storage capacity) and ^11^C-dihydrotetrabenazine (DTBZ, vesicular monoamine transporter function), or SPECT tracers such as ^123^I-β-CIT and ^123^I-FP-CIT [59,60]. ^123^I-FP-CIT SPECT imaging has demonstrated increased accuracy in differentiating SNCApathies, especially DLB, from AD in which DAT is preserved [58,61]. This ligand is mainly used in detecting the loss of nigrostriatal neuron terminals in patients with PD, but also in patients with clinically uncertain parkinsonian syndromes and individuals with essential tremor [62]. Therefore, DAT imaging is not useful in discriminating DLB from PD-MCI and PDD. In order to discriminate DLB patients from FTD or atypical parkinsonian syndromes (including progressive supranuclear palsy (PSP) and corticobasal degeneration (CBD)), ^123^I-FP-CIT-SPECT should not solely be accounted for as a reliable method of investigation [48]. 

Presynaptic dopaminergic metabolism is impaired in all basal ganglia disorders; therefore, presynaptic dopaminergic biomarkers cannot differentiate between parkinsonian syndromes. Nevertheless, ^123^I-FP-CIT SPECT imaging may be useful to distinguish MSA from other cases of cerebellar ataxias, especially in cases of onset in adult patients [63]. Postsynaptic D2/D3 receptor biomarkers show reduced binding in most patients with MSA, whereas they show a negative scan in PD [64]. The clinical phenotype should always be considered when interpreting findings, and integrated imaging modality should be considered in order to optimize the therapy approach.

### 4.2. Cardiac Sympathetic Innervation Imaging in SNCApathies

^123^I- Iobenguane (MIBG) cardiac scintigraphy is widely used to assess cardiac postganglionic sympathetic degeneration, which is a common feature in DLB and PD [58], in order to exclude AD in the differential diagnosis of DLB. In contrast to patients with PD, in whom cardiac postganglionic sympathetic innervation is reduced in virtually all cases, most patients with MSA have preserved postganglionic innervation of the heart [60,65], even if a minority of patients with MSA may have some degree of cardiac sympathetic denervation [66]. Therefore, in the differential diagnosis of PD vs. MSA, MIBG imaging evidence of intact cardiac sympathetic innervation almost certainly excludes PD, but does not exclude MSA [65].

^11^C-meta-hydroxy-ephedrine (^11^C-mHED) is a PET radiopharmaceutical, and it is a catecholamine analog used for the assessment of cardiac sympathetic function [67]; in comparison to ^123^I-MIBG SPECT, ^11^C-mHED PET has a higher spatial resolution (3–4 mm for PET vs. 8–10 mm for SPECT) with a shorter duration of the exam (owing to the short physical half-life of ^11^C of about 20 min). Despite these favorable features, further studies are required for the clinical use of ^11^C-mHED.

### 4.3. FDG-PET Imaging in SNCApathies

In patients with PD, brain FDG-PET is normal or shows an increase in uptake in the putamen nucleus [60]. FDG-PET should be performed, as its adds diagnostic value, in order to detect prodromal dementia with Lewy bodies. Therefore, it seems to be particularly helpful in differential diagnosis of MCI. Even if measures of accuracy are not available, the presence of preserved metabolism in the posterior cingulate area (cingulate island sign) and occipital hypometabolism at the stage of MCI support a diagnosis of DLB, even if DAT imaging or cardiac sympathetic innervation imaging should be sought as a more informative investigation. Furthermore, FDG-PET imaging is an essential tool in the differential diagnosis between DLB vs. AD and DLB vs. FTD [13]. 

In patients with MSA, specific hypometabolic patterns have been described such as reduced uptake in both putamen nuclei with a rostrocaudal gradient [64]. Decreased FDG-PET uptake can also be detected in the thalamus, brainstem, and cortical areas. Therefore, hypometabolism in the putamen nucleus, mesencephalic region, and cerebellum is a supportive feature for MSA [68].

### 4.4. Amyloid Imaging in SNCApathies

The relationship between Aβ pathology and dementia in LB disorders to date is unclear but in DLB, apart from α-synuclein aggregation, the neurological changes may also include amyloid-β and tau deposition [69]. Therefore, amyloid-β and tau deposition, assessed by functional imaging, could support the differential diagnosis between AD-related pathology and α-synucleinpathies. ^11^C-Pittsburgh compound B (^11^C-PiB) imaging studies have reported reduced retention in DLB when compared to patients with AD, but increased retention in DLB when compared to patients with PD or PDD [70,71]. DLB subjects have higher amyloid burden than subjects with PDD, PD-MCI, or PD or controls. Furthermore, the higher burden leads to greater cognitive impairment in DLB. Early amyloid deposits in DLB relative to PDD may account for their difference in the timing of dementia and parkinsonism [72]. Therefore, amyloid imaging will have an upgraded role when anti-amyloid treatments are available for DLB patients.

### 4.5. Tau Imaging in SNCApathies

Concerning in vivo tau deposition evaluation, cortical ^18^F-AV-1451 uptake was more significant in DLB than in the controls or cognitively normal PD, especially in the inferior temporal gyrus and precuneus, where increased binding resulted associated with cognitive impairment. Nevertheless, ^18^F-AV-1451 PET uptake was more extensive and severe in AD compared to DLB patients, allowing the discrimination of DLB from AD. Further studies have been performed in order to evaluate the integrated role of amyloid and tau deposition in DLB, reporting that medial temporal lobe AV-1451 uptake distinguishes AD dementia from DLB, and that ^11^C-PiB uptake is associated with greater posterior temporoparietal and occipital ^18^F-AV-1451 uptake, indicating an atypical pattern of molecular deposition in DLB [73,74]. Further studies are needed in order to assess the relationship between amyloid and SNCApathies, but amyloid imaging remains a promising tool.

### 4.6. Alpha-Synuclein Imaging in SNCApathies

The development of a reliable SNCA radioligand could provide a valuable tool, especially when combined with other modalities, and, to date, remains a challenge of neuroimaging in synucleinopathies. Several potential compounds with acceptable characteristics have been identified, such as benzoxazole ^18^F-BF227, which shows a high affinity for amyloid and low affinity for SNCA in postmortem brain tissues from MSA patients [75,76]. Furthermore, a series of 3-(benzylidine)indolin-2-one derivatives were synthesized and evaluated for their in vitro binding to alpha-synuclein (α-syn), beta-amyloid (Aβ), and tau fibrils, and resulted in increased binding to α-syn and reasonable selectivity for α-syn versus Aβ and tau [77]. A further preclinical and clinical trial is needed, but alpha-synuclein imaging seems particularly promising to understand the full spectrum and progression of overlapping proteinopathies, which may lead to novel therapeutic approaches.

## 5. Other Atypical Parkinsonian Syndromes

The term atypical parkinsonian disorders refers to a heterogeneous group of neurodegenerative movement disorders, such as multiple system atrophy and dementia with Lewy bodies but also progressive supranuclear palsy (PSP) and corticobasal degeneration (CBD). The parkinsonian syndrome is accompanied in all these diseases by many additional clinical findings not typical for PD such as rapid progression, gaze palsy, apraxia, ataxia, early cognitive decline, dysautonomia, and usually low response to levodopa.

### 5.1. FDG-PET Imaging in Atypical Parkinsonian Syndromes

CBD is a less common tauopathy with clinical presentations that include cortical dysfunction, basal ganglia dysfunction, and nonresponsiveness to levodopa. In CBD, pathological changes are mainly localized predominantly in the frontoparietal cortex, basal ganglia, and substantia nigra [78]. Corticosubcortical atrophy (contralateral to the side of the body mainly affected) may be detected by regional cerebral blood flow imaging with SPECT or PET unilateral hypoperfusion [79]. 

In PSP, the neuronal degeneration affects many areas of the brain: midbrain, superior cerebellar peduncle, subthalamus, pallidum, dentate nucleus, and frontal lobes, with subsequent radiological abnormalities [80], particularly the atrophy of the midbrain called the hummingbird sign [81]. PSP SPECT and FDG-PET imaging allows the evaluation of a typical hypoperfusion/hypometabolism pattern in these regions, especially frontal lobes striatum, thalamus, and midbrain [82]. The EANM panel supports the use of FDG-PET in the differential diagnosis among several forms of dementia, including PSP from PD. Nevertheless, FDG imaging shows moderate sensitivity in differentiating PSP from CBD and MSA. The hypometabolism in CBS is mainly described in motor and premotor cortices, but can also involve the prefrontal or posterior parietal and lateral temporal cortex, and the cingulate gyrus. This heterogeneity is consistent with the variety of diseases causing CBS, which mainly include CBD, PSP, AD, FTLD, or combination of pathologies. Nevertheless, cases of a prevalent parietal lobule and posterior cingulate hypometabolism can be considered consistent with AD, whereas cases of hypometabolism mainly localized in the anterior cingulate and associated frontal cortex can be considered consistent with PSP or FTD. Moreover, a basal ganglia hypometabolism supports CBD and PSP [13]. 

### 5.2. DAT Imaging in Other Atypical Parkinsonian Syndromes

DAT imaging has prognostic value in determining whether patients with parkinsonism and behavioral and/or language dysfunction will develop features of PSP or FTD later in the disease course [81]. DAT imaging with PET or SPECT, in fact, can be used to support or refute a diagnosis of dopamine-deficient parkinsonism in cases where this is unclear, with the implication of rationalizing a trial of dopamine therapy. The presence of normal DAT availability on imaging can help to categorize subjects without evidence of dopamine deficiency from patients with reduced levels of baseline striatal DAT availability on PET or SPECT scanning, which should be considered as supportive rather than diagnostic of dopamine-deficient parkinsonism [83].

### 5.3. Tau Imaging in Other Atypical Parkinsonian Syndromes

Considering the physiopathology of these diseases, tau imaging can be a useful tool. Further trials are needed, but, according to previous papers, patients with CBS exhibited AV-1451 retention in the motor cortex, corticospinal tract, and basal ganglia contralateral to the affected body side, clearly different from controls and patients with AD dementia or PSP [84]. Furthermore, ^18^F-THK5351 is considered as a promising candidate radiotracer for the in vivo imaging of tau deposits in CBS [85].

However, cortical atrophy measured with MRI and decreased ^18^F-fluorodeoxyglucose uptake were more widespread than ^18^F-AV-1451 uptake and probably represented earlier, yet less specific, markers of CBS. Furthermore, in PSP, tau imaging with a 2-(1-{6-[(2-^18^F-fluoroethyl)(methyl)amino]-2-naphthyl}ethylidene)malononitrile ^18^F-FDDNP probe allows the detection of tau aggregates in subcortical and cortical areas with a typical pattern of PSP and may be a valuable diagnostic tool for all PSP phenotypes or a prognostic biomarker [86]. According to a previous paper, in PSP and PD, distinct subcortical ^18^F-AV-1451 binding patterns have been described, reflecting subcortical tau pathology in PSP and reduced nigral neuromelanin in PD. Nevertheless, the authors described no correlation with the severity of motor dysfunction and no cortical regions with increased binding in PSP patients [87]. Furthermore, ^18^F-THK-5351 PET can potentially be used to detect the regional tau distribution in brain regions in PSP, and therefore this novel radiopharmaceutical may potentially support the differential diagnosis of neurodegenerative disorders associated with tau protein. In fact, ^18^F-THK5351 PET signals reflect MAO-B expressing reactive astrocytes [88], which may be associated with tau accumulation in PSP.

## 6. Summary of Techniques Validated for Clinical Practice

In Europe, the “European Association of Nuclear Medicine and European Academy of Neurology recommendations for the use of brain ^18^F-fluorodeoxyglucosepositron emission tomography in neurodegenerative cognitive impairment and dementia” panel voted to support the use of FDG-PET in clinical practice whenever a neurodegenerative disorder is suspected to be the underlying cause of an MCI condition, including the conditions of AD, FTD, and DLB. The use of FDG-PET is not supported in any of the preclinical conditions, suggesting the use of this tool for research purposes, including asymptomatic conditions in patients at risk for AD. However, this does not exclude the application of imaging in selected cases to answer specific personalized questions nor its use in clinical trials. Moreover, the panel supports the use of FDG-PET in clinical practice in prodromal and dementia stages of most neurodegenerative disorders due to its ability to detect the location and extension of neuronal dysfunction even at early stages [13].

According the “SNMMI (Society of Nuclear Medicine and Molecular Imaging) Procedure Standard/EANM Practice Guideline for Amyloid PET Imaging of the Brain 1.0”, in Europe the use of amyloid PET is considered appropriate when: the patient has persistent or progressive unexplained MCI; the core clinical criteria for possible AD are satisfied but there is an unclear, atypical, or mixed clinical presentation; or the patient has progressive dementia and the age of onset is ≤65 y. Nevertheless, these guidelines should be updated in the case of possible future anti-amyloid therapies, and further health services research is necessary to determine the effective clinical use of amyloid PET [89].

The indication of the EANM practice guideline/SNMMI procedure standard for dopaminergic imaging in parkinsonian syndromes supports the use of presynaptic dopaminergic imaging in Europe for detecting loss of nigrostriatal dopaminergic neuron terminals in patients with parkinsonian syndromes, especially: in the differential diagnosis between essential tremor and neurodegenerative parkinsonian syndromes; in the differential diagnosis between DLB and other dementias; or in the differential diagnosis between parkinsonism due to presynaptic degenerative dopamine deficiency and other forms of parkinsonism, in early presynaptic parkinsonian syndromes.

Moreover, postsynaptic dopaminergic imaging can help separate typical from atypical parkinsonian syndromes where loss of D2 receptors occurs (e.g., MSA, PSP) [90]. However, these indications and guidelines do not exclude the application of imaging in selected cases for a personalized approach nor its use for research purposes. 

Further studies are needed to assess the role of tau imaging in clinical practice in Europe. Nevertheless, tau imaging remains a promising tool, especially in the case of possible future anti-tau therapies.

## 7. Conclusions

Molecular imaging technologies are playing a crucial role in neuroimaging by providing a “window” into the living brain. Where CT and conventional MR imaging provide essential structural and anatomical information on the brain, molecular imaging technologies allow physicians to evaluate brain functionalities. Nuclear medicine imaging has evolved from being a tool used in the research field in order to investigate neurological diseases (dementia but also other neurological conditions, including primary or secondary neoplasms, or further neurological disorders, such as multiple sclerosis) to being a standardized imaging tool used in neurological clinical practice. In recent years, new software and techniques have been developed in order to improve the quality of the images acquired and, subsequently, in order to maximize the clinical impact of these techniques. In addition, recent development in the radiopharmaceutical field has allowed nuclear medicine imaging to be increasingly used in clinical practice and research regarding dementia, by exploring multiple physiopathological aspects of diseases including glucose metabolism, tau and amyloid retention, etc. The role of nuclear medicine in the differential diagnosis of dementia is crucial as it leads to a personalized approach to dementia. Moreover, both standardized and novel radiopharmaceuticals (once validated) can be considered as progression biomarkers and as surrogate markers for monitoring the efficacy of therapy.

## Abbreviations

AD	Alzheimer’s disease
FTD	Frontotemporal dementia
SNCApathies	The alpha-synucleinopathies
